# Computed Tomography in Limb Salvage and Deformity Correction—3D Assessment, Indications, Radiation Exposure, and Safety Considerations

**DOI:** 10.3390/jcm10173781

**Published:** 2021-08-24

**Authors:** Lukas Zak, Thomas M. Tiefenboeck, Gerald E. Wozasek

**Affiliations:** Department of Orthopedics and Trauma-Surgery, Trauma Surgery, Medical University of Vienna, Waehringer Guertel 18-20, 1090 Vienna, Austria; thomas.tiefenboeck@meduniwien.ac.at (T.M.T.); gerald.wozasek@meduniwien.ac.at (G.E.W.)

**Keywords:** CT, deformity correction, distraction osteogenesis, external ring fixation

## Abstract

Computed tomography (CT) is an essential tool in orthopedic surgery but is known to be a method with that entails radiation exposure. CT increases the risk of developing fatal cancer, which should not be underestimated. However, patients with bone defects and/or deformities must frequently undergo numerous investigations during their treatment. CT is used for surgical planning, evaluating callus maturation, alignment measurement, length measurement, torsion measurement, and angiography. This study explores the indications in CT scans for limb lengthening and deformity correction and estimates the effective radiation dose. These results should help avoid unnecessary radiation exposure by narrowing the examination field and by providing explicit scanning indications. For this study, 19 posttraumatic patients were included after the bone reconstruction of 21 lower limbs. All patients underwent CT examinations during or after treatment with an external ring fixator. The mean effective dose was 3.27 mSv, with a mean cancer risk of 1:117,014. The effective dose depended on the location and indication of measurement, with a mean dose of 0.04 mSv at the ankle up to 6.8 mSv (or higher) for vascular depictions. CT evaluation, with or without 3D reconstruction, is a crucial tool in complex bone reconstruction and deformity treatments. Therefore, strict indications are necessary to reduce radiation exposure—especially in young patients—without compromising the management of their patients.

## 1. Introduction

Injuries of the lower limb are common in high-energy traumata and are often accompanied by open fractures with large bone defects and soft tissue injuries [1]. In these cases, various radiological evaluation methods, such as conventional radiography, computed tomography (CT), (color flow) Doppler ultrasound, digital subtraction angiography, and computed tomography angiography (CTA) are crucial. These methods may be necessary for emergencies, complication assessments, presurgical planning, and decision-making in bone reconstruction [2].

Computed tomography has become the method of choice to detect bone pathologies and lesions as well as subtle or non-displaced fractures, in instances where conventional radiographs have limitations [3]. The last decade’s developments enabled the acquisition of three-dimensional data sets with a submillimeter spatial resolution, facilitating the creation of high-speed detailed image reconstruction and display techniques [2].

Spiral CT in-plane slides, or 3D reconstructions, have become the preferred imaging modality for orthopedic patients with various indications [4]. This method offers a variety of advantages, such as fast scan time, high resolution, and good availability [5]. The rapid generation of 3D data sets can immediately display the results on the screen, enabling quick interpretations and demonstrations for clinicians [5]. In emergency cases, CTA is a reliable technique for traumatic arterial injury diagnosis in the extremities [6]. This method also helps medical professionals select patients for interventional radiologic procedures [2] and plan microsurgical reconstructions [7].

High-resolution and powerful computers are necessary for 3D postprocessing and 3D reconstruction in trauma and orthopedic surgery. This method’s areas of application include posttraumatic intraosseous rotation of the scaphoid, [8] planning in maxilla-fascial surgery, [9] angiographic depictions, [10] angiographic planning for soft tissue flaps, [11] and planning for complex injuries [4] or various pathological conditions masked by metal artifacts [4,12].

This study aimed to present the various indications for CT scans in limb salvaging, bone reconstruction, and deformity correction. To our knowledge, no published study on this topic has estimated the effective dose of radiation or the risk of developing fatal cancer. The discussion of differences between deformities, indications, locations, ages, and genders highlights the necessity of safe and effective imaging.

We hypothesized that patients with lower limb deformities would receive higher radiation exposure during treatment than healthy patients, resulting in a highly increased risk for induced fatal cancer.

## 2. Patients and Methods

### 2.1. Patients

All procedures involving human participants followed the ethical standards of the institutional ethical review board (Nr. 1054/2016), along with the 1964 Helsinki Declaration and its later amendments (or comparable ethical standards).

For this retrospective study, we reviewed our departmental database for patients with posttraumatic bone defects or shortening of the bone. At our Trauma Department, between 2006 and 2015, 25 patients underwent bone reconstruction surgeries and limb lengthening via treatment with an external ring fixator for bone lengthening, bone transport, and axis correction. This study included 19 patients with 21 treated extremities evaluated by CT scans for planning and follow-up. Both open and closed fractures were primarily or secondarily treated with a Taylor Spatial Frame (TSF, Smith and Nephew, Memphis, TN, USA) (19 extremities) or an Ilizarov frame (2 extremities). Patients suffered from motor vehicle accidents (MVAs), motorcycle accidents (MCAs), industrial accidents (IAs), falls from a great height, gunshot fractures (GSFx), or other accidents. These traumas resulted in bone loss, bone shortening, pseudarthrosis, bone infection, or axial deviation. The patient characteristics and pre-history are presented in Table 1 and a list of all patients in Table 2. Six patients were excluded, as no CT scans were performed during their observation periods. Further exclusion criteria included pediatric or adult non-traumatic deformity correction, as well as patients treated by intramedullary lengthening.

Patients were evaluated during emergency care, preoperatively after initial damage control orthopedic (DCO) treatment (CT angiography, torsion measurement), during treatment for the evaluation of callus maturation to assess appearance, and during the healing of pseudarthrosis to measure alignment (or for follow up).

### 2.2. CT Evaluation

We used two different CT Scanners during the measurement period: a Siemens Somatom (Sensation Open, 64 slices, Siemens, Forchheim, Germany) and a Siemens Sensation 4 (Siemens, Forchheim, Germany).

Images were read on our PACS System (IMPAX EE, 2018 Agfa-Gevaert Group, Mortsel, Belgium) using the Volume Rendering Technique (VRT) software (IMPAX EE, 2018 Agfa-Gevaert Group, Mortsel, Belgium) employed for multidimensional image processing and computer viewing (in some cases).

When CT angiography of the pelvis and lower extremities was performed, an intravenous contrast agent (OptirayTM 350 mg J/mL, MallinckrodtTM Pharmaceuticals, Hennef, Germany) of 120 mL was applied.

The overall image quality was graded as excellent, good, fair, or poor, as adapted from Adibi et al. [2]. 

### 2.3. Radiation Exposure

The Effective Dose (ED) in millisieverts (mSv) was calculated by multiplying the dose-length product (DLP) by the body-region-specific conversion coefficient k: “E = k ∗ DLP” [13]. DLP was determined from the protocol of every CT scan, and the coefficient “k” was used as a specific weighting factor for the tissue, evaluated on a phantom model considering the scanned region and the patients’ ages and genders [14].

Based on the ED, patient age, and patient gender, the estimated risk of radiation-induced fatal cancer (RFC) can be measured. For this purpose, we used the probability coefficient of an adult population with an age range of 25 to 64 years (mean 45 years), [15] according to the 1990 Recommendations of the International Commission on Radiological Protection (ICRP), [16] which was updated in 2007 [17]. The RFC corresponds to 1 in 25 patients after exposure to 1 Sv (1000 mSv) [15].

For individual gender- and age-related patient RFCs, we used the nominal probability coefficients for stochastic radiation effects of the ICRP, as presented in Cross et al. [15].

### 2.4. Indications

#### 2.4.1. CT Angiography

**Figure 1:**
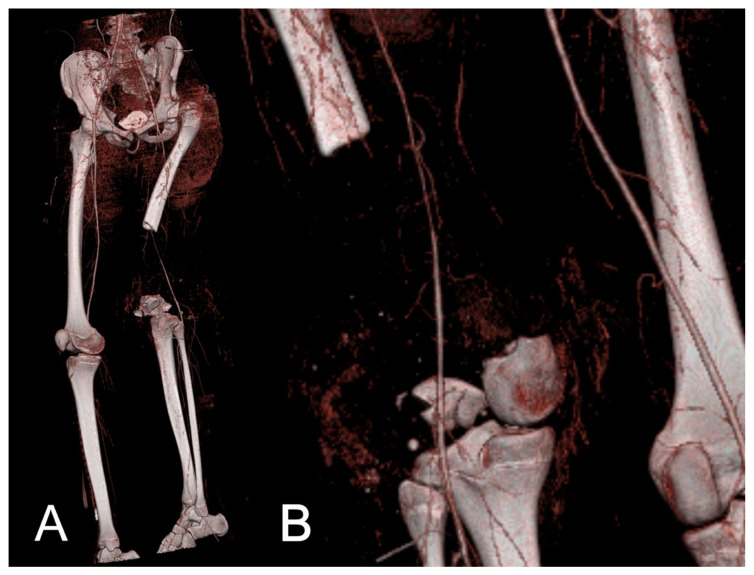
Massive bone defect in a 15-year-old patient on the left distal femur. Angiography was performed to decide between reconstruction and amputation and to plan the surgery. Oblique–anterior view. (**A**) The femoral artery has a reduced caliber but remains intact with a continuous flow. (**B**).

CT angiography (CTA) or VRT was indicated for presurgical planning after dislocated fractures or significant bone defects to show vascular injury in axial views, in order to decide between limb salvage and amputation based on the course of the large vessels. An example is shown in Figure 1. Another indication was to follow-up after vascular repair and other vascular interventions to detect vascular stenosis or narrowing.

#### 2.4.2. Non-Union Evaluation

**Figure 2:**
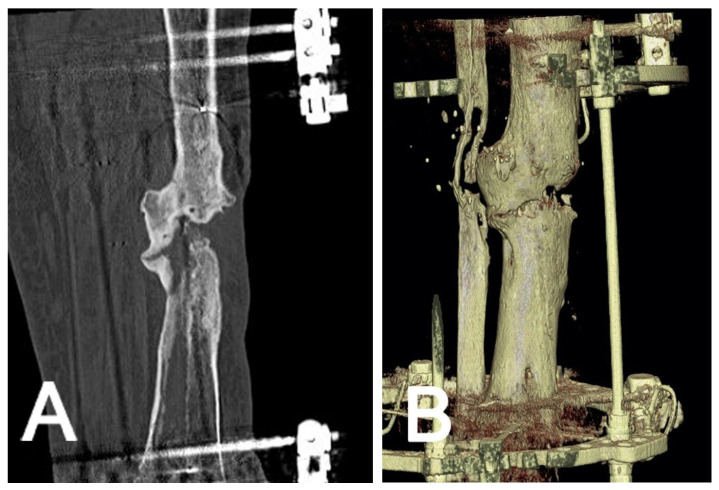
Non-union evaluation in a tibial bone with a 2D (**A**) and 3D view (**B**).

For pseudarthrosis and non-union evaluation, a CT scan is essential for further planning. A helpful tool for pseudarthrosis and non-union evaluation is VRT, which can provide a three-dimensional representation of the bone (Figure 2). In this method, the illustrated metal parts of the external frame or cast can be removed from the picture to better understand the region of interest. The callus patency can then be evaluated, and the decision to engage in fixator removal can be made when three cortices are healed [18].

#### 2.4.3. Axis and Alignment

**Figure 3:**
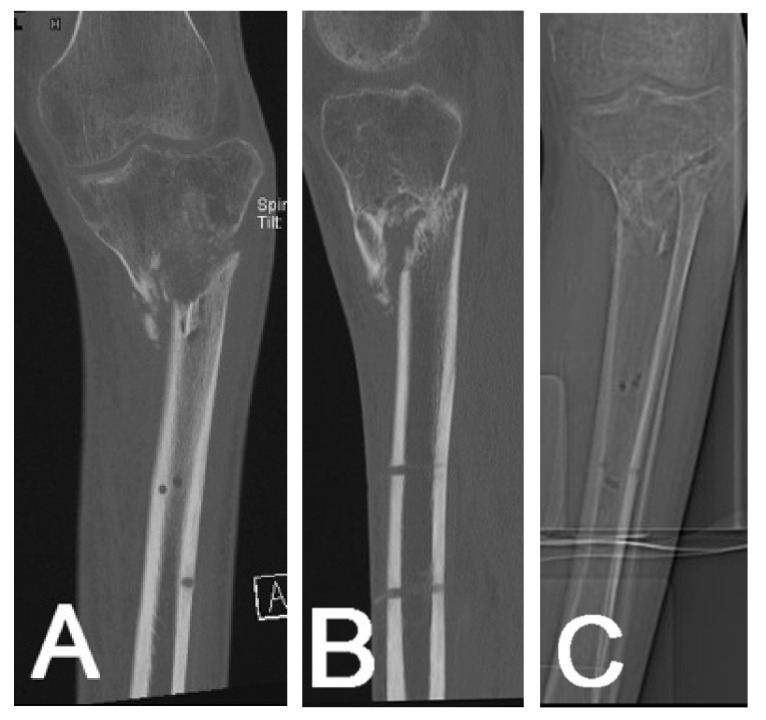
Conventional CT scan to plan deformity correction in a displaced proximal tibial fracture—2D AP view (**A**) and lateral view (**B**) showing translation and varus deformity. Scanogram from the same patient in AP view (**C**).

For axis and alignment measurements, CT scans with 2D and 3D representations are able to provide images of the bone from all angles (Figure 3). Correction planning in simple external fixators or ring fixators can then be performed, and planning schedules can be adapted according to the new data.

#### 2.4.4. Surgical Planning

**Figure 4:**
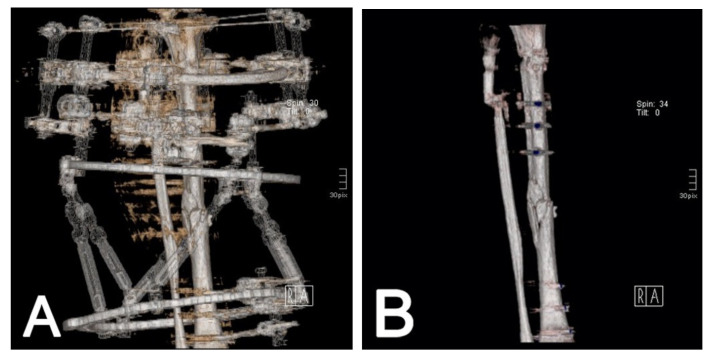
Three-dimensional reconstruction of the bone for planning. Artifacts and fixators can interfere with visibility in the region of interest (**A**) but can be removed by the software (**B**).

Besides the planning of non-union treatment and axis correction, further interventions were planned through CT and VRT. Presurgical evaluation of complex fractures was performed to plan ring fixator assembly and axial malalignment. For this purpose, the amount of bone loss was illustrated in 2D or 3D for different strategies (Figure 4). CT scans can support the decision to engage in fixator removal if three intact cortices are visible [18], as measured on two planes in the 2D view or depicted in the VRT. To evaluate callus maturation, in some cases, external frames or casts were removed by the software (Figure 4B).

#### 2.4.5. Leg Length Measurement

**Figure 5:**
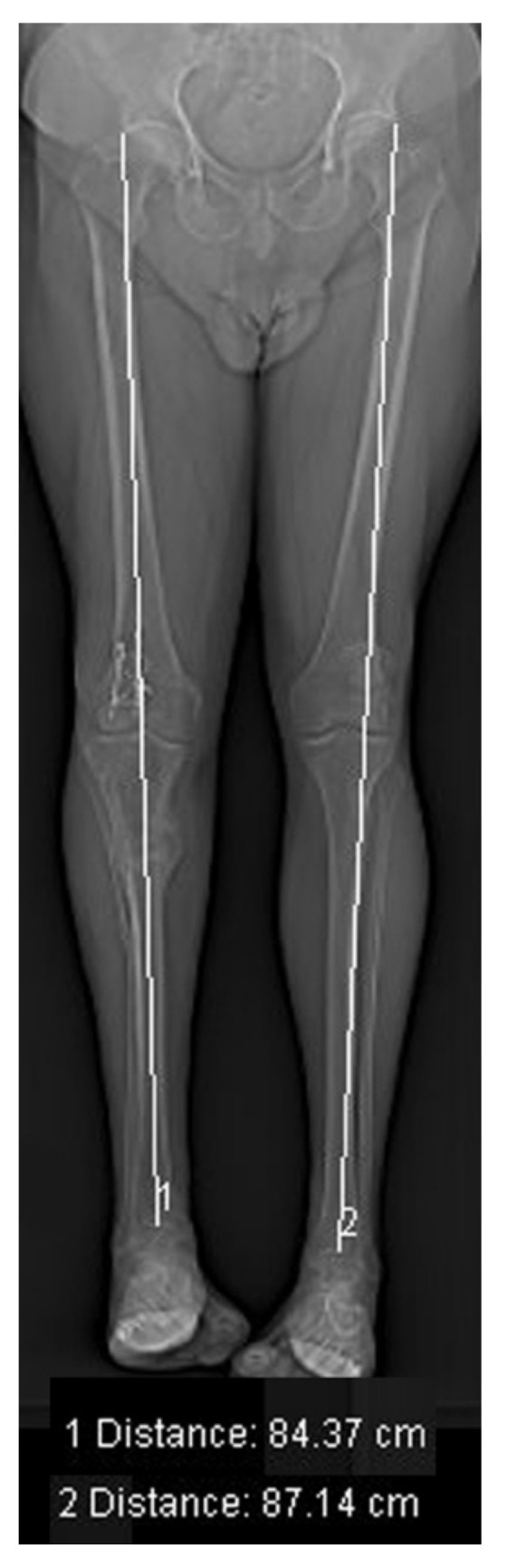
Length measurement of the lower limb to plan bone-lengthening surgery. The measurement shows a 2.8 cm LLD.

The leg length was measured using a CT scanogram [19]. (Siemens Sensation Open, Figure 5) in lieu of the conventional alignment views commonly employed in the scanogram technique [19]. (Philips Medical System Digital Diagnost). This method facilitated more precise evaluations for presurgical planning. The correct length for customized arthrodesis nails or implants was measured via this method, and limb length discrepancies were evaluated to determine the distraction schedule.

#### 2.4.6. Rotational CT/Torsion CT

**Figure 6:**
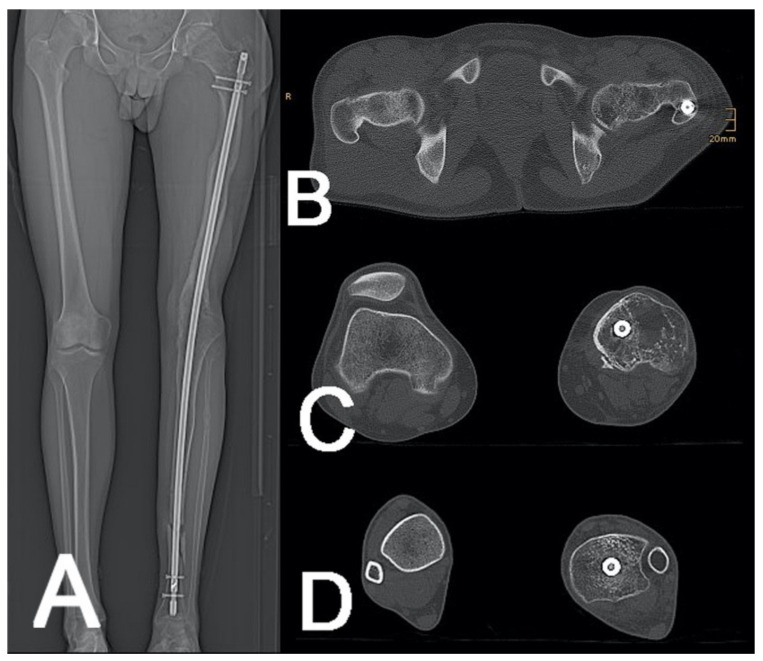
Rotational CT to plan the de-rotation surgery of a knee arthrodesis nail (**A**). Measurements of the hip (**B**), knee (**C**), and ankle joint (**D**) were performed.

For rotational alignment measurement/lower limb torsion measurement, three acquisition zones were defined on an anterior–posterior (ap) scout view including the hip, knee, and ankle (Figure 6).

Femoral and tibial torsion measurements are usually performed using axial CT images [20]. to evaluate the rotational malalignment between the injured and non-injured extremity.

## 3. Results

Treatment decisions were made according to the depictions of vessels, types of fracture, alignments, callus maturation, and rotation. Table 3 shows the distribution of the scans, specific questions, and indications. In total, 34 CT scans were performed on 19 patients. In 12 out of 23 conventional CT scans and 6 out of 8 angiographic CT scans, a VRT-reconstruction helped to answer specific questions for posttraumatic deformity correction. In most cases, more than one problem was analyzed for further therapeutic planning.

The results for the patients’ effective doses and risk of fatal cancer are presented in Table 5, showing a mean risk of 1 in 213,920 per patient, based on the individual probability coefficient, or 1 in 117,014 for an average adult patient. These vast differences are a result of age and gender differences. A 2.3-fold risk for the youngest patients and 20% risk for the oldest patients were observed.

The ED depends on the indication of measurement and the location. The mean ankle measurement for an average adult person starts at 0.04 to 0.12 mSv and rises to 6.8 mSv or higher for vascular depictions in CTA. Here, the RFC (1 in 25 per 1 Sv) increased from 1 in 700,000 to 1 in 5000 (Table 4 and Table 5).

The mean quality, according to the classification of Adibi et al., was 3.4 for the CT scans and 3.1 for the VRT presentation. In 88.2% of CT measurements and 75% of VRT measurements, the image quality was graded as good to excellent, and external fixators or casts did not influence accessibility. The CTA quality was graded as 3.3 and the CTA VRT quality as 3.0, indicating at least good diagnostic potential for arterial injuries.

## 4. Discussion

The present work aimed to describe indications for CT scans in limb reconstruction and deformity correction based on a case study evaluating the quality and dose effects in various indications. Furthermore, this paper examined the risk of radiation exposure among this specific patient population. To our knowledge, no other study in this field has analyzed or highlighted this issue.

CT bone scanning of the extremities has a significantly higher effective dose than that of conventional radiography but entails considerably lower radiation exposure than investigations of the trunk region. [14,15].

For conventional x-rays of the extremities, an average effective dose of 0.001 to 0.005 mSV per series is expected [21]. In contrast, a CT-scan of the lower limbs created amounts of 0.0013 (CT ankle joint) to 6.63 mSV (CT angiography of a female) in our series, depending on the modality and issue.

Alternative methods that use no radiation, such as ultrasound and MRI, are available with limitations. Indeed, as most modern commercial fracture fixation implants do not contain any ferromagnetic materials, magnetic resonance imaging (MRI) is commonly used as an alternative assessment method for long bones [22,23]. The advantage of MRI is its ability to depict soft-tissue abnormalities adjacent to the pathology [24]. Ultrasound can also be used to evaluate callus maturation but is disadvantageous in detecting angulation [25].

Both methods do not have the ability to create 3D reconstructions. The volumetric rendering technique (VRT) enhances the visualization of pathologies and helps to handle large data sets. VRT effectively shows subcortical lesions, minimally displaced fractures, and hidden areas of interest [10]. This technique can reduce visible streak artifacts, even in the presence of metal implants, pins, or prostheses. [4,10]. Various practical algorithms and reconstruction techniques are also available [26]. The most common indications are CTA (Figure 1), non-union evaluation (Figure 2), and surgical planning (Figure 4). In emergency situations, a standard procedure has been developed to detect and characterize traumatic arterial injuries of the extremities using CTA [6]. This procedure is non-invasive and allows one to measure different areas of the body simultaneously [6]. CT has also been used to determine fracture stability [27] and rotational malalignments [28,29]. and seems to be superior to conventional radiographs for non-union evaluations, as CT is unaffected by abundant calluses or the presence of a cast [3]. However, CT’s almost-100% sensitivity contrasts with the low specificity of other techniques (62% to 83%) [30]. Complementary methods such as SPECT/CT scans are available with lower sensitivity but reasonable specificity that excludes infection and confirms the non-union site’s nonviability [31].

For length measurements, CT scanograms (0.6 mS) were described as being more accurate and requiring fewer radiation doses than computed radiography (CR) scanograms, with three large exposures used for the hip, knee, and ankle (2 mSv) in a pediatric patient population [19]. CR can capture the entire femoral and tibial length and minimize measurement errors [19,32]. Sabharwal et al. evaluated 111 patients with LLD using a CR-based scanogram and teleoroentgenogram. Ultimately, 4.6% (33 mm) magnification was measured for the lower extremity’s absolute length using a standing radiograph, with a mean difference of 5 mm in the LLD measurement between the two CR techniques [19,33]. Among our patients, we discovered two faulty presurgical planning cases using conventional long axial views. We replaced this method in individual cases with a CT topogram (0.19 mSv), showing discrepancies between 2.9% and 3.7%. These differences can lead to severe consequences in the planning and performance of orthopedic surgery.

For our risk calculations, the calculated effective dose was based on the measured DLP and the *k*-values of a phantom model, with the disadvantage of not considering the individual patient-specific habitus [14]. The scanner was from the same company (in both studies) [14,34], as we expected comparable values. The effective dose depends on the scanner, scanning area, and gender. [14,17].

Cross et al. [15] reported a radiation exposure of 0.5 mSv for knee and foot/ankle measurements, whereas Koivisto et al. presented values of 0.021 mSv, which are comparable with our results (Table 4). The ED of the pelvic CT was reported with a mean value of 8 mSv [35]. and the hip scan with a mean value of 3.09 mSv. These results refer to multiple doses of foot and lower-leg scans, with EDs of 0.16 mSv (knee) and 0.07 mSv (ankle) [36]. These values are comparable to those in our study, which combined knee and knee/proximal lower leg (0.14 mSv) or ankle and ankle/distal lower leg (0.08 mSv) measurements (Table 4).

The risk for inducing fatal cancer was calculated as 1 in 25 per 1 Sv (1000 mSv) for an adult worker to simplify measurements for the indication and location-specific statements. For patient-specific measurements, age and gender were also considered. The risk is more than twice as high for children and teenagers and about half as high for elderly patients around 70 years of age [15]. For adult women, the tissue-weighting factors and the risk of fatal cancer are the same as those for men for all tissues, except the breast [15].

In one case (Nr. 3), seven CT scans (mostly CTA) were performed to follow up on a vascular lesion with an individual high cancer risk of 1 in 1649 for a 70 year-old female patient. Nearly the same risk (1 in 1856) was measured for an adolescent patient with two necessary CTs (CTA and a hip–knee–ankle CT for rotational measurement), with a 5.6-fold higher risk per SV.

According to Kovacs et al., exposure during chest, abdominal, and pelvic examinations could be reduced by up to 50% by adapting protocols over a period of 5 years (2010–2015)—values that cannot be achieved for extremity scans [37]. However, recent technological advances and refined protocols have been developed to reduce radiation doses. A low-dose CT protocol for the purpose of surgical planning can strongly reduce radiation exposure compared to traditional CT without affecting image quality or diagnostic performance [38,39]. Contrast enhancement seems to be valuable only in the evaluation of soft-tissue structures [40]. As metal implants increase radiation exposure, [41] external ring fixators with carbon rings (TSF) and a higher amount of non-metal parts are advantageous compared to full metal devices.

The limitations of this study include its small patient number, retrospective design, and the absence of a control group without cast or metal devices.

In a few cases, metal artifacts severely influenced the CT or VRT evaluations of fracture or callus maturation. Metal implants not only deteriorate image quality but also increase radiation exposure [41]. This was not considered in our measurements, as measurements typically took place before metal implantation, and deformity correction was typically performed with a TSF.

MRI can serve as an alternative method entailing less radiation exposure and using compatible non-ferromagnetic external fixators with fewer noise artifacts [23]. under safe conditions [42]. Furthermore, in the present study, each particular scan time was not evaluated separately.

## 5. Conclusions

Patients with complex bone lesions and deformities may undergo numerous diagnostic medical investigations. CT scanning with or without 3D reconstruction is, therefore, a crucial tool and can be necessary to handle complications, to decide fixator removal, and/or to assess non-unions. CT is commonly applied with acceptable radiation exposure in various indications. However, medical professionals should remain aware of high radiation exposure in angiographic CT measurements of the pelvis and lower limbs. These measurements result in an increased risk of fatal cancer, especially in children, adolescents, and young adults. Therefore, the number of scans and regions of interest must be limited to the exact indications of CT examinations to protect patients from radiation absorption. This paper is intended to promote awareness and help orthopedic surgeons decide upon the use of CT scans for deformity correction, thereby avoiding unnecessary radiation exposure or pitfalls.

## Figures and Tables

**Table 1:** Patient characteristics, mean ± SD (range); M, male; F, female; TSF, Taylor Spatial Frame; MVA, motor vehicle accident; tib–fib, tibial–fibular.

Patient Characteristics	
**N**	19 patients 21 extremities
**Demographic data**	
Age (years)	44 ± 20 (15–82)
M	37 ± 15.6 (15–73)
F	61 ± 19.5 (27–82)
Sex (M/F)	13/6
**Baseline characteristics**	
Ring fixator/TSF	2/19
**Trauma**	19 patients
MVA	11
Industrial accident	2
Fall	4
Gunshot accident	2
**Deformity**	
Pseudarthrosis	4
Bone defect/shortening	5
Axial deviation	4
Axis + shortening	5
Osteomyelitis	1
**Location**	21 extremities
Open tib–fib **fracture**	14
Open femur fracture	1
Prox tibial fracture	2
Ankle Fracture	1
Tib–fib Gunshot fracture	3

**Table 2:** List of all patients, including age, gender, location on the lower limb, trauma anamnesis, type of fracture, and treated type of deformity.

Patient’s List						
	Age	Sex	Location	Accident	Fracture	Treatment
1	49	m	tib–fib	MVA/MC	open Fx	PA, bone transport Ilizarov
2	40	m	tib–fib	MVA/MC	open Fx	PA, bone transport TSF over nail
3	70	f	tib–fib	Fall	open Fx	PA, axial deviation, vessel lesion, TSF
4	18	m	tib–fib	Industrial a.	open Fx	bone defect, shortening, Ilizarov
5	34	m	tib–fib	MVA	open Fx	bone defect and soft tissue, PA, TSF
6	36	m	tib–fib/tib–fib	Gunshot	GSFx/GSFx	left bone defect and refracture TSF, right axial deviation
7	45	m	tib–fib	MVA/MC	prox tib Fx	axial deviation and shortening, TSF
8	50	m	tib–fib	MVA	prox tib Fx	axial deviation and shortening, TSF
9	15	m	tib–fib+femur	MVA/MC	open Fx	huge bone defect, shortening, bone transport and lengthening, TSF
10	73	m	tib–fib	Gunshot	GSFx	axial deviation and shortening, TSF
11	32	m	tib–fib	MVA/MC	open Fx	bone defect and shortening, TSF
12	31	m	tib–fib	IA.	open Fx	shortening, TSF
13	32	m	tib–fib	MVA	open Fx	axial deviation and shortening, TSF
14	27	f	tib–fib	MVA (ped.)	open Fx	deux etage, healing in TSF
15	20	m	tib–fib	Fall	open Fx	osteomyelitis and fistulation, TSF
16	67	f	tib–fib	Fall	ankle Fx	axis correction and arthrodesis TSF
17	50	f	tib–fib/tib–fib	Fall	open Fx	axial deviation and shortening, TSF
18	82	f	tib–fib	MVA (ped)	open Fx	axial deviation, TSF
19	70	f	tib–fib	MVA/MC	open Fx	axial deviation, TSF

F—female; m—male; MVA—motor vehicle accident; MC—motorcycle; tib–fib fx—tibial and fibular fracture; IA—industrial accident; PA—Pseudarthrosis; ped.—pedestrian; FfH—fall from height; TSF—Taylor Spatial Frame.

**Table 3:** List of all measurements, including the scans and indications for the scans; LLD, Limb length discrepancy.

Scans and Indications	
Scans	
Total	34
CT	23 (12 VRT)
Hip	2
Knee	3
Knee + lower leg	4
Lower leg	8
Lower leg + Ankle	3
Ankle	3
Angio CT	8 (6 VRT)
Rotational CT	2
CT scanogram	1
Specific questions/indications	48
Vessels	8
Non-union	10
Axis	4
Pre-/Further surgical planning	9
Fixator removal/Callus maturation	5
Length measurement, LLD	5
Malrotation	7
Mixed Indications	13
SD Standard deviation	

**Table 4:** Patient list with measured effective dose (ED), risk of induced fatal cancer (RFC) per Sv (1000 mSv), individual patient-specific risk, and risk for an adult person (1 in 25 per Sv).

Effective Dose and RFC—Patients							
	Age	Sex	Nr. CTs	ED (mSv)	RFC Per Sv	RFC (Individual)	RFC (Adult)
1	49	m	2	2.21	1 in 24	10,860	11,312
2	40	m	2	0.16	1 in 24	151,052	157,346
3	70	f	7	37.60	1 in 62	1649	665
4	18	m	1	4.95	1 in 11	2222	5051
5	34	m	1	5.15	1 in 23	4468	4856
6	36	m	3	2.72	1 in 23	8464	9200
7	45	m	1	0.19	1 in 24	129,450	134,844
8	50	m	2	0.28	1 in 24	86,331	89,928
9	15	m	2	5.93	1 in 11	1856	4217
10	73	m	1	0.11	1 in 59	561,905	238,095
11	32	m	1	0.34	1 in 23	67,548	73,421
12	31	m	2	0.22	2 in 23	102,679	111,607
13	32	m	2	0.13	3 in 23	172,673	187,688
14	27	f	1	0.29	1 in 14	48,110	85,911
15	20	m	1	0.12	1 in 16	130,081	203,252
16	67	f	1	0.06	1 in 34	570,470	419,463
17	50	f	4	1.44	1 in 26	18,038	17,344
18	82	f	1	0.10	1 in 143	1,471,193	257,202
19	70	f	1	0.12	1 in 62	525,424	211,864
				3.27	1 in 34	213,920	117,014

**Table 5:** Effective Dose (ED) and risk of fatal cancer (RFC, 1 in X) in various indications and locations for an average adult worker.

Effective Dose and Risk of Fatal Cancer		
Specific Questions/Indications	Mean ED	RFC
Vessels (CTA)	6.80	3677
Non-union	0.49	51,502
Axis	0.20	123,793
Pre-/Further surgical planning	0.29	87,054
Fix. Removal/Callus maturation	0.10	250,479
Length measurement, LLD	0.38	66,246
Malrotation	2.34	10,701
Location		
CTA	5.04	4956
Topo	0.19	134,844
Hip	2.54	9825
Knee	0.10	262,608
Knee + lower leg	0.21	118,147
Lower leg	0.18	140,417
Lower leg + Ankle	0.12	217,050
Ankle	0.04	692,252

ED, Effective dose; LLD, Limb length discrepancy.

## Data Availability

For this retrospective study, we reviewed our departmental database for patients with posttraumatic bone defects or shortening of the bone.

## Abbreviations

CT	Computer Tomography
CTA	Computer Tomography Angiography
MVA	Motor vehicle accident
MCA	Motorcycle accident
IA	Industrial accident
GSFx	Gunshot fracture
TSF	Taylor Spatial Frame
DCO	Damage Control Orthopedics
tib–fib	tibial–fibular
Fx	Fracture
PA	Pseudarthrosis
ped.	Pedestrian
m	male
f	female
ED	Effective Dose
DLP	dose length product
VRT	Volume Rendering Technique
LLD	Limb length discrepancy
mSV	Millisievert
MRI	Magnetic Resonance Imaging
